# A Child with Acute Encephalopathy Associated with Quadruple Viral Infection

**DOI:** 10.3389/fped.2015.00026

**Published:** 2015-04-02

**Authors:** Keiko Nakata, Mitsuru Kashiwagi, Midori Masuda, Seiji Shigehara, Chizu Oba, Shinya Murata, Tetsuo Kase, Jun A. Komano

**Affiliations:** ^1^Department of Infectious Diseases, Osaka Prefectural Institute of Public Health, Osaka, Japan; ^2^Department of Paediatrics, Hirakata City Hospital, Hirakata, Japan; ^3^Department of Clinical Laboratory, National Hospital Organization Nagoya Medical Center, Nagoya, Japan

**Keywords:** acute encephalopathy, coxsackievirus A6, enterovirus D68, human parechovirus, human herpesvirus-6, risk factor, viral co-infection

## Abstract

Pediatric acute encephalopathy (AE) was sometimes attributed to virus infection. However, viral infection does not always result in AE. The risk factors for developing infantile AE upon virus infection remain to be determined. Here, we report an infant with AE co-infected with human herpesvirus-6 (HHV-6) and three picornaviruses, including coxsackievirus A6 (CVA6), Enterovirus D68 (EV-D68), and human parechovirus (HPeV). EV-D68 was vertically transmitted to the infant from his mother. CVA6 and HPeV were likely transmitted to the infant at the nursery school. HHV-6 might be re-activated in the patient. It remained undetermined, which pathogen played the central role in the AE pathogenesis. However, active, simultaneous infection of four viruses should have evoked the cytokine storm, leading to the pathogenesis of AE. *Conclusion:* an infant case with active quadruple infection of potentially AE-causing viruses was seldom reported partly because systematic nucleic acid-based laboratory tests on picornaviruses were not common. We propose that simultaneous viral infection may serve as a risk factor for the development of AE.

## Introduction

Acute encephalopathy (AE) is a neurological dysfunction that presents with altered consciousness, sometimes associated with neurological injury or death in young children (1, 2). Many etiologic factors have been identified, including infectious agents and genetic factors. AE in young children is often associated with virus infection. The risk factors for developing infantile AE upon virus infection remain to be determined because infections with the aforementioned viruses usually cause mild symptoms.

Acute encephalopathy in young children is often associated with virus infection, including influenza virus (3–5), human herpesvirus-6 (HHV-6) (6), rotavirus (7), mumps virus (8), and human parechovirus (HPeV), a newly discovered virus of the *Picornavirus* family (9). One of the most systematic etiologic studies on AE, in which 983 subjects were examined, was performed in Japan during 2007–2010 (10). The three most frequently detected pathogens were human influenza virus (HIFV, 26.6%), HHV-6 (17.0%), and rotavirus (4.0%). Other pathogens were respiratory syncytial virus (RSV), human adenovirus (HAdV), and HPeV. Only five cases showed co-infection with two pathogens, such as HHV-6 and RSV or rotavirus and *Campylobacter jejuni*. Etiology was undetermined for approximately half of the infant AE cases. Such a study has not been performed in developing countries.

Laboratory technology for detecting pathogens at the nucleic acid level is advancing, and this may provide clues as to the etiology of AE. Here, we describe an infant AE case that may provide insights into this question.

## Background

### Patient history

**Figure 1:**
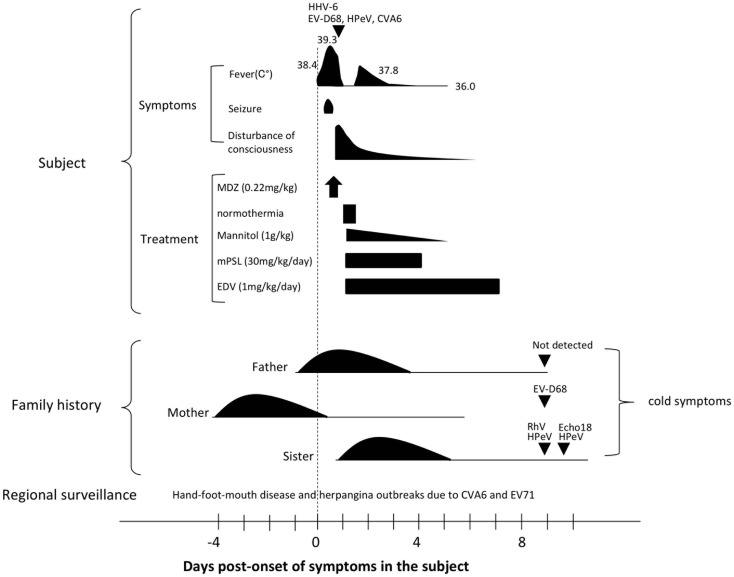
**Clinical course of the presented subject and his family history**. The triangle indicates the day of specimen collection for the subject. Detected pathogens were indicated above each triangle. Days post-onset refer to the time-course of symptoms in the subject. Please see the main text for details. Abbreviations: HHV-6, human herpesvirus type-6; EV68, enterovirus 68; HPeV, human parechovirus; CVA6, coxsachievirus A6; MDZ, midazolam; mPSL, methylpredonisolone; EDV, edaravone; RhV, rhinovirus; and Echo18, echovirus 18.

A previously healthy male infant aged 1 year and 9 months presented to a primary physician with fever (38.4°C). During a physical examination, the infant had a convulsive seizure accompanied by flaccid paralysis of the limbs, upward tonic eye deviation, and tachycardia (190 beats/min). The subject was admitted to an emergency hospital. On arrival, the subject presented with high fever (39.3°C) and status epilepticus (Figure 1).

### Laboratory examination, imaging, and electroencephalography

Laboratory examinations revealed a white blood cell count of 19,060 cells/ml (neutrophils, 51.1%; lymphocytes, 43.4%; and monocytes, 4.0%); red blood cell count of 4.42 million cells/ml; and blood hemoglobin level of 9.5 g/dl. Most of the serum markers remained within the normal range, including C-reactive protein (CRP), total bilirubin (TB), creatine phosphokinase (CPK), blood urine nitrogen (BUN), and creatinin. Serum levels of aspartate aminotransferase (AST), alanine aminotransferase (ALT), and lactate dehydrogenase (LDH) were slightly elevated. Blood sugar was 240 mg/dl. Serum pro-inflammatory markers interleukin-6 (IL-6) and interleukin-1 beta (IL-1b) were 299 pg/ml (normal range, <4.0 pg/ml) and 1.4 pg/ml (normal range, <10 pg/ml), respectively. Cerebrospinal fluid (CSF) was colorless and transparent. CSF glucose, blood cell count, and total protein were within normal ranges. CSF IL-6 was 40 pg/ml, and IL-1b was undetectable. A cranial computed tomography scan and magnetic resonance imaging of the brain without contrast showed no particular findings. An electroencephalogram demonstrated diffuse, high-amplitude slow wave activity.

### Examination of infectious agents

The blood HHV-6 DNA load was 3 × 10^2^ copies/ml, but HHV-6 was not detected in CSF. The plasma titer for anti-HHV-6 IgG was 1:60, and IgM was undetectable.

Enterovirus species were further examined because of the family history (detailed below) and a hand–foot–mouth disease (HFMD) outbreak in the nursery school where the subject was attending. In addition, regional surveillance revealed HFMD and herpangina outbreaks due to coxsackievirus A6 (CVA6) and enterovirus 71 (EV71) infections. Nucleic acid-based examination and virus isolation tests in tissue culture and suckling mice were performed (11). Enterovirus D68 (EV-D68) was detected in throat swab and stool specimens. HPeV was also positive for RNA extracted from the throat swab (12). Additionally, CVA6 was positive in the stool. A tissue culture-based virus isolation test detected HPeV-1 from the stool specimen (13). In the suckling mice-based virus isolation test, CVA6 was detected in stool specimen (14). However, CVA6, EV71, and EV-D68 were not detected by nucleic acid-based examination in CSF.

Beside these viruses, immunochromatographic tests were negative for rotavirus, HAdV, and RSV on admission. Nucleic acid-based tests were negative for HHV-7, Japanese encephalitis virus (JEV), vesicular stomatitis virus (VSV), and herpes simplex virus type (HSV) in blood and CSF. Bacterial cultures from blood and CSF were negative.

In summary, four viruses were detected: HHV-6 and three picornavirus species, namely, EV68, HPeV-1, and CVA6 (Figure 1; Table 1).

**Table 1:** **Summary of viruses detected in the patient, his family, and regional surveillance**.

Subject	Detected virus	Specimen
Patient	CAV A6	Stool
	EV-D68	Throat swab, stool
	HHV-6	Blood
	HPeV	Throat swab, stool
Mother	EV-D68	Stool
Father	Not detected	–
Older sister	Echo18	Stool
	HPeV	Throat swab, stool
	RhV	Throat swab
Regional surveillance	CVA6	HFMD and herpangina patients
	EV71	HFMD and herpangina patients

*CAV, coxsackievirus; Echo18, echovirus 18; EV71, enterovirus 71; EV-D68, enterovirus D68; HFMD, hand–foot–mouth disease; HHV-6, human herpesvirus-6; HPeV, human parechovirus; RhV, rhinovirus*.

### Family history

Four days prior to onset of illness in the infant, his mother presented with cold symptoms. A day before and after the onset of illness in the infant, his father and older sister, aged 3 years and 4 months, also presented cold symptoms. PCR-based laboratory tests revealed that stool specimens from the mother were positive for EV-D68. Three enterovirus species were detected from his older sister: the stool specimen was positive for echovirus 18 (Echo18) and the throat swab was positive for both HPeV and rhinovirus (RhV). No virus was detected from the patient’s father (Figure 1; Table 1).

## Discussion

### Diagnosis, treatment, and outcome

The differential diagnosis included acute encephalitis, acute meningitis, and non-infectious causes of AE such as toxic or metabolic encephalopathy. We diagnosed this patient as AE associated with active infection of four viruses because of the low inflammatory markers. The seizure lasted 52 min, and was stopped by intramuscular administration of midazolam (0.22 mg/kg). The subject was then administered mannitol infusion 1 g/kg, methylprednisolone 30 mg/kg/day, and edaravone 1 mg/kg. Therapeutic normothermia (36.0°C) for 12.5 h was also performed. One day post-admission, the subject regained consciousness. However, drowsiness remained until 7 days post-admission. No apparent sequelae were recognized at discharge (Figure 1).

### Risk factor of acute encephalopathy

This is the first report of four potentially AE-causing viruses detected in an infant with AE (15–18). The subject did not take vaccine and travel abroad recently. The regional surveillance and the family history suggest that EV-D68 was vertically transmitted to the infant from his mother. Considering epidemiologic factors, CVA6 and HPeV were likely transmitted to the infant at the nursery school. Thus, the infant had been infected with three picornaviruses at once. Given that none of the four viruses were found in all of the specimens from the patients, their viral loads were low compared with viral loads in primary mono-infections partly because replication of the picornaviruses *in vivo* could have been limited by viral interference (19). Regarding HHV-6, the viral load was relatively low and viral DNA was not detected in the CSF. In addition, plasma IgM against HHV-6 was undetectable. These data indicate that HHV-6 has already established a latent infection although the possibility of HHV-6 primary infection is not eliminated. Simultaneous co-infection with the three picornaviruses could have damaged the immunity of this subject, leading to partial immunosuppression. This might have triggered a reactivation of latently infected HHV-6. The transient immunosuppressive state with active infection of four viruses might have evoked a physiological state akin to a so-called cytokine storm, which can cause AE (1, 15, 20, 21). Thus, simultaneous infection of viruses might be a risk factor for disease severity, most likely stemming from picornavirus species. These hypotheses require experimental verification in future studies.

## Concluding Remarks

Picornavirus is often overlooked as an etiological factor for AE. We successfully detected three picornaviruses because we carefully monitored the regional surveillance data and used established, systematized laboratory examination protocols. Laboratory technology to detect pathogens at the nucleic acid level is advancing, and this may provide clues as to the etiology of AE.

## Author Contributions

KN performed the laboratory examinations, drafted, and reviewed the manuscript. MK, MM, SS, CO, and SH examined the subject of interest at the medical institution, performed medical practices, and collected clinical information and specimens. TK and JK coordinated the study. JK supervised data collection, drafted, reviewed, and finalized the manuscript. All authors approved the final manuscript as submitted.

## Conflict of Interest Statement

The authors declare that the research was conducted in the absence of any commercial or financial relationships that could be construed as a potential conflict of interest.
